# Using a Visual Turing Test to Evaluate the Realism of Generative Adversarial Network (GAN)-Based Synthesized Myocardial Perfusion Images

**DOI:** 10.7759/cureus.30646

**Published:** 2022-10-24

**Authors:** Akinori Higaki, Yoshitaka Kawada, Go Hiasa, Tadakatsu Yamada, Hideki Okayama

**Affiliations:** 1 Department of Cardiology, Ehime Prefectural Central Hospital, Matsuyama, JPN; 2 Department of Cardiology, Pulmonology, Hypertension & Nephrology, Ehime University Graduate School of Medicine, Toon, JPN; 3 Department of Intractable Disease and Aging Science, Ehime University Graduate School of Medicine, Toon, JPN

**Keywords:** digital literacy, deepfake, myocardial perfusion imaging, generative adversarial networks, visual turing test

## Abstract

As the quality of image generation by deep learning increases, it is becoming difficult to discern its authenticity from the image alone. Currently, generative models represented by generative adversarial networks (GAN) are increasingly utilized in the research field of cardiology, and their potential risks are also being pointed out. In this context, we assessed whether expert cardiologists can detect synthesized myocardial perfusion images (MPI) generated by GAN as fake. A total of 1448 polar maps collected from consecutive patients who underwent MPI for known or suspected coronary artery disease from January 2020 to December 2021 were used for the analysis. A deep convolutional GAN was trained on the polar maps to synthesize realistic MPI. The realism of the generated images in terms of human perception was evaluated by the visual Turing test (VTT) on our original website. The average correct answer rate of the VTT was only 61.1% with a standard deviation of 21.5, but this improved to 80.0±15.8 (%) in the second trial when given the clue information. In the era of machine intelligence and virtual reality, digital literacy is becoming more necessary for healthcare professionals to identify deepfakes.

## Introduction

As the quality of image generation by deep learning increases, it is becoming difficult to discern its authenticity from the image alone. Currently, generative models represented by generative adversarial networks (GAN) are increasingly utilized in the medical domain [1], and their potential risks are also being pointed out [2,3]. One of the most important risks to be aware of is the possibility of misdiagnosis due to the attenuation of features in medical images [4]. Another risk includes the malicious use of artificial intelligence (AI)-based deepfake technology [5]. Recently, Thambawita and colleagues have reported that a realistic electrocardiogram could be synthesized by deepfake technologies [6]. Although the authors positively interpret their results as the end of privacy issues in medicine, the same result can be seen as the beginning of the confusion unless the generated data are distinguishable from the real objects. However, the importance of this issue does not seem to be fully recognized in the field of cardiology. In this context, the present study assessed whether expert cardiologists can detect synthesized myocardial perfusion images (MPI) generated by GAN as fake. Herein, we also introduce how we implemented a visual Turing test (VTT) as a web application.

## Technical report

Image data collection

A total of 1448 polar maps collected from consecutive patients who underwent MPI for known or suspected coronary artery disease from January 2020 to December 2021 were used for the analysis. Stress/rest thallium-201 (Tl-201) myocardial perfusion scintigraphy was performed according to the American College of Cardiology/American Heart Association/American Society of Nuclear Cardiology clinical guidelines for cardiac radionuclide imaging [7]. The polar maps in stress and rest were extracted from the single-photon emission computed tomography (SPECT) studies as color images in JPEG format and rescaled to 64x64 respectively for the subsequent analysis. Details of the data collection are described in our previous literature [8].

Polar maps synthesized by generative adversarial networks

**Figure 1:**
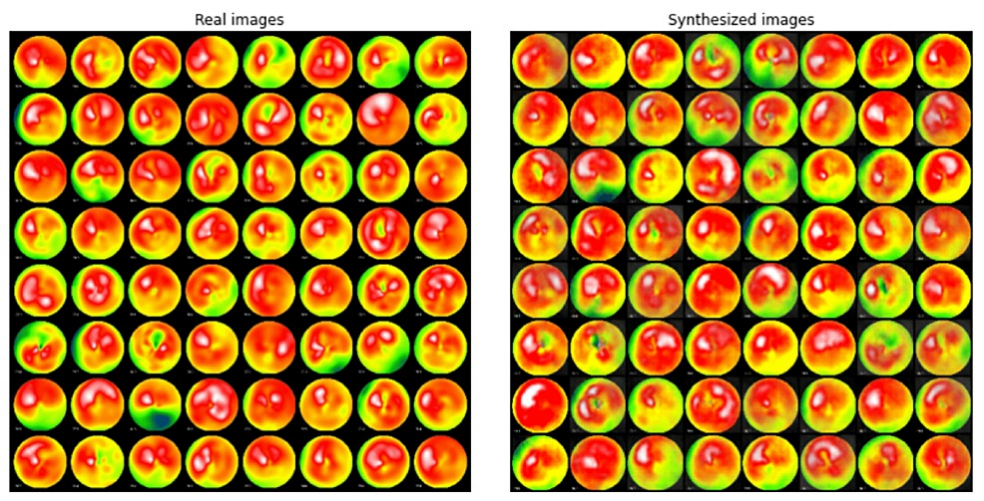
Comparison of real and synthesized MPI polar maps. The left panel shows examples of real polar maps obtained from patients. The right panel shows examples of synthesized polar maps by GAN. The FID between the original and generated data was computed as 100.6. The BRISQUE score of the original images and generated images were 49.9±8.0 and 32.3±7.5, respectively. GAN: generative adversarial networks; FID: Fréchet inception distance; BRISQUE score: Blind/Referenceless Image Spatial Quality Evaluator score

The original implementation of deep convolutional GAN (DCGAN) was trained on the collected image data [9]. In this study, both stress and rest images are used as training data. The DCGAN model was constructed referencing the “PyTorch DCGAN Tutorial (https://pytorch.org/tutorials/beginner/dcgan_faces_tutorial.html)”, but the batch normalization layers were removed from the discriminator network to regulate its performance. Figure 1 shows the example of real (true) and fake (synthesized) MPI polar maps.

Evaluation of the image generation performance

Fréchet inception distance (FID) between the original and generated data was computed as 100.6. The Blind/Referenceless Image Spatial Quality Evaluator (BRISQUE) score was significantly higher in the original images than in generated images (49.9±8.0 vs 32.3±7.5, p<0.001 by Welch's t-test).

**Figure 2:**
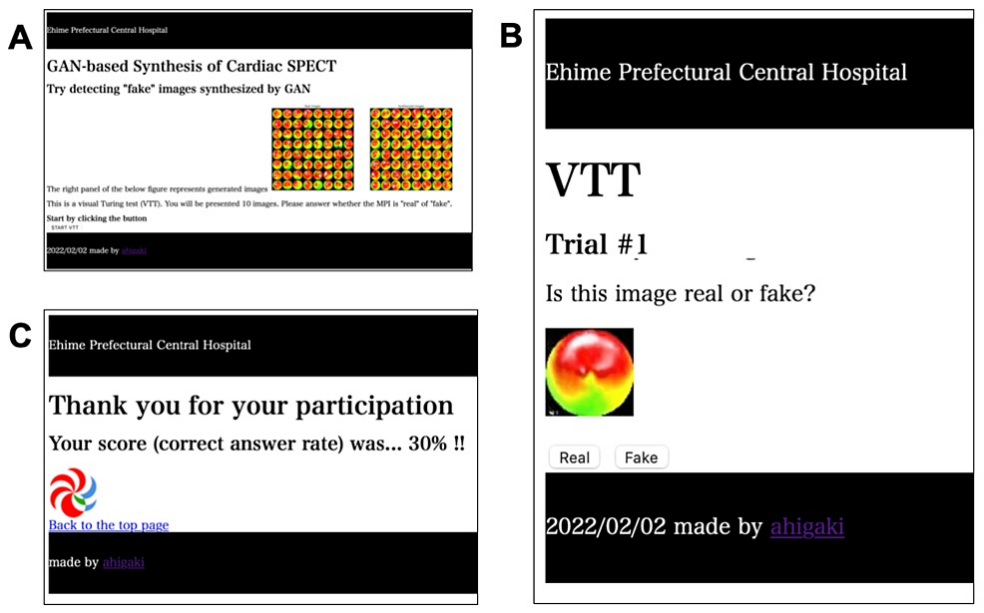
Screenshots of the web application of VTT. A web application was implemented on Glitch with JavaScript + HTML. On the top page, participants are presented with examples of generated and real images, as well as an explanation of the VTT methodology (panel A). Once the test starts, participants are presented with the polar maps one by one and prompted to answer whether the image is real or fake, as shown in Panel B. Each time one of the buttons is clicked, the presented image is randomly selected from a database containing a mixture of original and generated images. After a total of 10 questions, the CAR is calculated and displayed as shown in Panel C. CAR: correct answer rate

The realism of the generated images in terms of human perception was evaluated by the VTT [10]. Nine cardiologists (15.8±11.9 years of professional experience) certified by the Japanese Society of Cardiology participated in this test through a web application (https://visual-turing-test.glitch.me/). The participants were independently presented with a total of 10 bull’s eye images from the mixed dataset and asked to tell if the presented images were real or fake (Figure 2). 

As for the initial evaluation, the average correct answer rate (CAR) of the VTT was 61.1% with a standard deviation of 21.5, which was not significantly higher than random guess (vs 50%, p=0.16 by Welch's t-test).

After a six-month interval, the same VTT was performed on the evaluators who were informed of the characteristics of the synthesized images; they were a bit blurred in color. When the evaluators were informed of the characteristics of the synthesized image, the CAR improved to 80.0±15.8 (%), which was significantly higher than random guess (vs 50%, p<0.01 by Welch's t-test). This improvement was statistically significant (p=0.01 by paired-samples t-test).

**Table 1:** Results of VTT by 9 cardiologists The CAR of the initial trial was not significantly higher than that of a random guess. Significant improvement in CAR was observed in the second trial after a six-month interval. CAR: correct answer rate.

	Initial trial	Second trial	P value
CAR (%)	61.1 ± 21.5	80.0 ± 15.8	0.01

Data availability

The minimal dataset to reproduce our study results is accessible through a public repository (https://data.mendeley.com/datasets/mjhhw3zdwv/1).

## Discussion

Risks in medical applications of deep generative models can be intentional or unintentional. The former is called malicious tampering and is increasingly recognized as a potential danger in radiology [11,12]. On the other hand, unintentional modification of images, such as the latter, is also a problem that cannot be overlooked. Therefore, we believe that healthcare professionals should recognize these issues and act accordingly.

This report assessed whether skilled cardiologists can distinguish synthesized MPI polar map images using VTT, which we implemented as a web application. As a result, the average CAR of the participants was not significantly higher than a random guess. This result is in line with the recent study by Skandarani et al. reporting that imaging experts’ CAR for identifying synthesized cardiac MRI or echocardiogram was no higher than 60% [13]. Thus, at the time of writing, the AI models are successfully fooling cardiologists. Because the physicians who participated in this experiment had no expertise in AI, they were not familiar with GAN-generated images, which may have contributed to the low CAR in this study. In fact, none of the nine participants knew about GAN at the beginning of the experiment. Interestingly, the synthesized polar maps shows a lower BRISQUE score than the real polar maps, meaning that humans perceive the synthetic image more naturally. Nevertheless, the fact that average CAR increased by about 20% on the second trial suggests that there is room for improvement through educational training in their discrimination skills.

There are several limitations to our study. First, we only generated polar maps and did not attempt to generate the original slices of MPI. Second, since we employed a classical DCGAN in this study, the CAR could have been lower if we had used a modern GAN or diffusion model, which can produce higher-resolution images [14]. Third, the relatively small number of images presented to the evaluator in the VTT may have affected the CAR. In any case, caution is still required when using the generated medical images in clinical practice.

## Conclusions

This report demonstrated that GAN could synthesize realistic medical images that even skilled cardiologists could not detect as fakes. On the other hand, the results of repeated VTTs suggested that participants' knowledge of the AI may improve their discriminative ability. In the era of machine intelligence and virtual reality, digital literacy is becoming more necessary for healthcare professionals to identify deepfakes.
